# Effect of exercises with weight vests and a patient education programme for women with osteopenia and a healed wrist fracture: a randomized, controlled trial of the OsteoACTIVE programme

**DOI:** 10.1186/s12891-015-0811-z

**Published:** 2015-11-14

**Authors:** K. A. Hakestad, M. K. Torstveit, L. Nordsletten, M. A. Risberg

**Affiliations:** Department of Orthopaedic Surgery, Norwegian Research Center for Active Rehabilitation (NAR), Oslo University Hospital, Trondheimsveien 235, 0514 Oslo, Norway; Faculty of Health and Sport Sciences, University of Agder, Kristiansand and Grimstand, Norway; Department of Sports Medicine, Norwegian School of Sport Sciences, Oslo, Norway; University of Oslo, Oslo, Norway

**Keywords:** Osteopenia, Quadriceps strength, Weight vest, Exercise, Rehabilitation

## Abstract

**Background:**

Exercise programmes have shown to be important for the prevention of fractures in patients with established osteoporosis. However, few studies have evaluated the effect of such programmes for women with low bone mineral density (BMD) (osteoporosis or osteopenia) who have already suffered a fracture. Studies have indicated that exercise programmes concentrating on muscular strength and dynamic balance have a positive effect on significant risk factors for falls such as quadriceps strength and balance. The aim of the present study was to assess the effect of a 6-month exercise programme and a patient education component (OsteoACTIVE) on quadriceps strength, BMD, dynamic balance, walking capacity, physical activity level and quality of life in postmenopausal women with osteopenia and a previous wrist fracture.

**Methods:**

Eighty postmenopausal women with low BMD and a healed wrist fracture were randomized to OsteoACTIVE (*n* = 42) (age 65.5, range 51.2–79.2 years) or patient education only (control group) (*n* = 38) (age 63.9, range 52.7–86.8 years). Follow-up was conducted after 6 months (end of intervention) and 1 year. Outcome measures included quadriceps strength, BMD, dynamic balance, walking capacity, physical activity level and quality of life.

**Results:**

Thirty-five participants (83 %) completed the OsteoACTIVE programme. Mean adherence to OsteoACTIVE was 87 % (range 48–100 %). Twenty-five participants (72 %) met the a priori goal of 80 % adherence to the program. No adverse events were reported. There were no significant differences between the two groups over the 1-year follow-up for any of the outcome measures.

**Conclusion:**

The OsteoACTIVE rehabilitation programme revealed no significant effect on quadriceps strength, BMD, dynamic balance, walking capacity or self-reported functional outcomes over the 1-year follow-up.

**Trial registration:**

NCT01357278 at ClinicalTrials.gov (date of registration2010-04-21).

## Background

Osteopenia or osteoporosis is a progressive disease of the skeleton characterized by low bone mass and micro-architectural deterioration of bone tissue resulting in an increased risk of fragility fractures [1]. Fragility fractures lead to physical disability, a great deal of pain, impaired quality of life (QoL), increased mortality and higher health-care cost [2–4]. A previous wrist fracture is a risk factor for future vertebral or hip fractures with a relative risk of 4.4 and 1.9, respectively [5]. Among those already who have sustained a fracture, 50 % will experience a new fracture within a ten-year period [6]. Furthermore, there is an increased tendency to fall in elderly patients with reduced muscle strength and balance [7].

Physical activity has shown to increase muscle strength and bone mineral density (BMD) [8]. Furthermore, physical activity provides improved muscle control, balance and co-ordination and reduces the risk of falls [8]. Active rehabilitation in the form of structured exercise programmes may be one of the most important factors for the prevention of fractures due to low BMD [9, 10]. Several RCTs have found positive effects of weight-bearing activities to reduce falls and fracture risk, as well as to maintain or increase BMD in postmenopausal women with low BMD [7, 9–13]. In addition, structured exercise programmes in postmenopausal women with low BMD have shown to improve QoL [14].

Progressive high-intensity resistance exercises have shown to improve physical function and muscle strength in older adults [15, 16]. Progressive loading during weight-bearing activities may be effective in improving BMD in patients with low BMD [17]. The dose-response relationship between loading and improvements in BMD is nevertheless unknown [17]. The use of weight vests during exercise in individuals with low BMD without a fracture, has to our knowledge been reported in two studies [18, 19]. These studies reported significant improvements in muscle strength and balance, but no change in BMD [18, 19]. Weight vests could be used to increase loading on the spine and the lower extremities, with the goal of increasing load to the skeletal system and improving muscle strength [20], in older adults with low BMD.

Most studies on exercise interventions have examined women with normal healthy bones or low BMD [11, 12], but very few studies have included women with low BMD *and* previous fractures [21–23]. To our knowledge, no studies have used weight vests in patients with low BMD and a recent fracture.

Therefore, the main aim of this study was to evaluate the effect after 1 year of a 6-month active rehabilitation programme with the use of weight vests and including a patient education programme (OsteoACTIVE) on quadriceps strength, BMD, dynamic balance, walking capacity, physical activity level and QoL in postmenopausal women with osteopenia and a previous wrist fracture.

## METHODS

### Participants

The study was a single-blinded, randomized controlled study (https://clinicaltrials.gov/ct2/ show/NCT01357278?term=NCT01357278&rank=1 reference number NCT01357278 (date of registration 2010-04-21). The participants were included from the Department of Orthopaedic Surgery and the Emergency Ward at the Oslo University Hospital in Norway. We included (1) postmenopausal women > 50 years of age, (2) diagnosed with low BMD (t-score < −1.5), (3) wrist fracture not older than 2 years and healed at inclusion (no plaster cast), and (4) domiciled in the Oslo region. They were excluded if they (1) had had hip- or vertebral fractures, (2) history of > 3 osteoporotic fractures, (3) problems/illness indicating that active rehabilitation was not advisable, (4) were moderately or intensely physically active for more than 4 h per week, (5) were unable to understand written or spoken Norwegian.

All participants received oral and written information about the study and signed informed consent. The study was approved by the Regional Medical Research Ethics Committee of South- East Norway (reference number 1.2005.82), and conducted in accordance with the Helsinki Declaration. The study has adhered to the CONSORT guidelines.

### Intervention

The OsteoACTIVE rehabilitation programme consisted of a 6-month exercise programme combined with a patient education component (OsteoINFO). The exercise programme consisted of 2 group exercise sessions and one home exercise session per week (in total 3 × 60 min/week). The exercise programme had a progression of intensity and types of exercises, and was based on an established model developed at the University of British Columbia, Canada (Osteofit) [22, 24], and a Danish model [23]. Briefly, the main component of the exercises consisted of strength, balance, coordination and core stability exercises including weight vests. The exercises included in the OsteoACTIVE programme have previously been published by our group [25]. The patient education component (OsteoINFO) was based on the programme entitled “Choises for Better Bone Health” [26], and was offered twice, and each session lasted for two hours in both groups. The main component of the OsteoINFO programme comprised *What is osteoporosis?, Risk factors for osteoporosis, Nutrition for bone health?, Fall prevention* and *General exercise guidelines.* Participants allocated to the control group, who only received the OsteoINFO, were requested not to alter their original lifestyle habits.

### Measurements

Isokinetic quadriceps strength was the main outcome measure and was examined with a Biodex 6000 isokinetic dynamometer (Biodex 3 System Pro, USA). Peak torque in Newton metres (Nm) at 60° and total work in Joules (J) at 180° per second were measured. We have previously reported reliability data for isokinetic muscle strength tests and found high inter- and intra-rater reliability (ICC 0.89–0.93) in postmenopausal women with osteopenia [27].

**Anthropometry** Body mass index (BMI) was calculated with weight/(height x height). Absolute and percentage fat, fat-free mass and BMD were measured with dual-energy X-ray absorptiometry (DXA) (GE Lunar Prodigy and ENCORE Version 11.2: GE Healthcare, Waukesha, WI). The minimal detectable changes in BMD have shown to be 0.4 g/cm^2^ at the lumbar spine and 0.2 g/cm^2^ at the hip [28]. The scanned areas were the hip, femoral neck and trochanter, lumbar spine and total body.

**Dynamic balance** was evaluated with the four square step test (FSST) identifying those who were at risk of falls [29]. Cut-off score > 15 s has been established for multiple fallers (2 or more falls within the last 6 months, and <15 s for non-multiple fallers (fewer than 2 falls within the last 6 months) in older adults > 65 years of age [29]. The FSST has been found to be reliable (ICC = 0.99) and has a sensitivity and specificity of 85 and 88–100 %, respectively [29].

**Walking capacity** was measured with the six-minute walk test [30]. It has been validated to measure functional capacity in older adults [30]. To demonstrate clinical relevance, an improvement of 54 m is required [31]. The level of perceived exertion was recorded using the Borg scale (score range, 6–20, with 6 indicating “very easy” and 20 “very exhausting”), and was used after the six-minute walk test [32]. An improvement of 2 units on the Borg scale has been considered to be significant [33].

**Physical activity level** was evaluated using the validated self-reported Physical Activity Scale for the Elderly (PASE) [34, 35]. The modified Norwegian version on a scale from 0 to 315 was used, where 0 represents not active and 315 represents extremely active [36].

**Health-related QoL** was evaluated using the Medical Outcomes Study 36-item Short-Form Health Survey (SF-36) [37]. The questionnaire is divided into 8 subscales, each scored on a scale from 0 to 100. SF-36 includes aspects of physical function, role limitations-physical, bodily pain, general health, vitality, social function, role limitations-emotional and mental health. Studies have reported high reliability and validity [38], and an improvement of 3–5 points has been considered clinically relevant [39].

Data were collected at baseline, at 6 months and at 1-year follow-up by an independent investigator who was blinded to treatment allocation. The participants were asked to record their leisure-time physical activity level in a training diary. Adverse events were recorded in the training diary and the medical record.

### Sample size calculation

The power calculations were based on the primary outcome, quadriceps strength. With a clinically relevant difference between the groups for quadriceps isokinetic strength test of 10 % from baseline to 6 months, and a standard deviation for quadriceps strength of 12 Nm [40], 30 patients were needed in each group in order to achieve a statistical power of 90 % and a significance level of 0.05. Allowing for drop-out, 40 patients were included in each group.

### Randomization and blinding

Block randomization with blocks of 6, using sealed envelopes in series, was prepared by the statistician. A research coordinator, who was not involved in the testing or intervention, opened sealed envelopes containing the randomization allocation and assigned subjects to the OsteoACTIVE group or control group, accordingly. The assessor was blinded to group allocation throughout the trial and analysis period.

### Data analysis

To evaluate between group differences, a linear mixed model (variance component structure with time and time*group as fixed effects and time as random effect intercept and slope) was used (IBM® SPSS® Statistics, version 20.0 (IBM Corp., Somers, New York, USA). Intention- to-treat analysis was used to compare the OsteoACTIVE group and the control group [41]. Significance level was set to 0.05. The data are presented as mean difference (95 % CI) between groups at 6 months and 1-year follow-up. The 6-month intervention, with 2 weekly supervised group exercise sessions, consisted of maximum 48 group exercise sessions. The acceptable adherence to the exercise group was set to 80 % attendance rate, which represented 38 group exercise sessions.

## Results

**Fig. 1:**
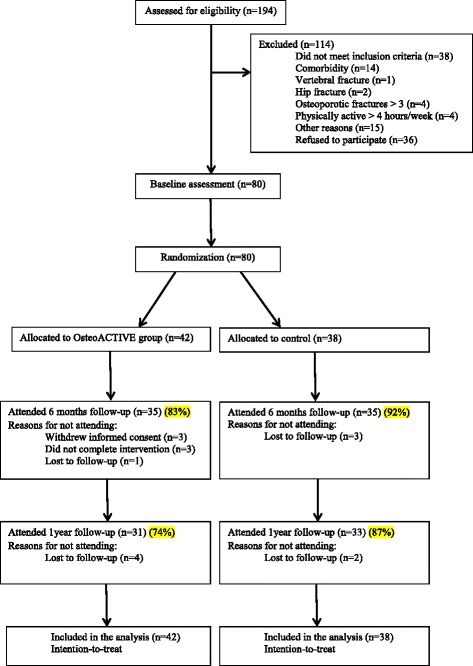
Flow-chart of the study

A total of 194 women were screened and eligible for participation, after which 80 women were enrolled and assessed (Fig. 1). Of the 80 women, 42 were randomly assigned to the OsteoACTIVE and 38 to the control group (Fig. 1). Of the 42 participants who were included in the OsteoACTIVE group, 3 withdrew informed consent prior to testing after the 6-month intervention. Total hip replacement, severe knee osteoarthritis and personal reasons prevented 3 other participants from completing the intervention programme. One additional participant was lost to follow-up, leaving 35 who completed the intervention programme (83 %). At 1-year follow-up, 4 participants were lost to follow-up (74 %) (*n* = 31) (Fig. 1). In the control group, 3 participants were lost to follow-up (92 %) (*n* = 35) at 6 months (Fig. 1). At 1-year follow-up, 2 additional participants were dropped out (87 %) (*n* = 33) (Fig. 1). The mean adherence rate for the 35 participants who completed the intervention was 87 % (range 48–100 %). Altogether 25 participants (72 %) met the a priori goal of 80 % adherence, while 10 participants (28 %) were below the 80 % attendance rate. No adverse events were reported by the participants in the OsteoACTIVE group.

The OsteoACTIVE and the control groups were similar at baseline with regard to age, height, weight, BMI, age of menopause, years since postmenopause, physical activity level and education (Table 1). The mean years since the time of fracture were 1.6 years (0.9) in the OsteoACTIVE group and 1.5 years (0.8) in the control group.

**Table 1:** Baseline characteristics of the participants

	OsteoACTIVE group	Control group
	(*n* = 42)	(*n* = 38)
Age (years)	65.5 ± 7.1	63.9 ± 7.1
Height (cm)	164.6 ± 6.3	164.4 ± 5.2
Weight (kg)	65.4 ± 10.6	66.2 ± 8.3
Body mass index (kg/cm^2^)	24.2 ± 4.1	24.3 ± 2.8
Body fat (kg)	23.4 ± 6.6	23.7 ± 6.6
Body fat (%)	36.4 ± 5.6	36.6 ± 6.1
Lean mass (kg)	39.7 ± 4.7	40.1 ± 3.9
Age of menarche (years)	13.3 ± 1.5	13.1 ± 1.7
Age of menopause (years)	48.7 ± 4.6	50.3 ± 4.1
Time since menopause (years)	16.7 ± 8.7	13.6 ± 8.3
Current use of bisphosphonate, n (%)	9 (21.4)	14 (36.8)
Current use of calcium, n (%)	2 (4.8)	2 (5.3)
Time since fracture by inclusion (years)	1.6 ± 0.9	1.5 ± 0.8
Past history of fracture (years)^a^	2 (1–3)	2 (0–3)
Family history of osteoporosis, n (%)	28 (66.7)	28 (73.7)
Current smoker, n (%)	4 (4.9)	5 (13.2)
Previous smoker, n (%)	13 (31)	12 (31.6)
Current alcohol use (4–7 units/week), n (%)	8 (19)	5 (13.2)
Educational attainment, n (%)		
Higher degree >3 (years)	14 (33.3)	14 (36.8)
Lower degree <3 (years)	28 (66.7)	24 (63.2)
PASE (0–315)^b^	103.1 ± 56.7	114.6 ± 58.7

Values are mean ± SD unless otherwise indicated

^a^Value is median (minimum-maximum)

^b^PASE, The Physical Activity Scale for the Elderly

Over the 1-year follow-up no difference was found between the OsteoACTIVE group and the control group for the main outcome quadriceps strength (Table 2). Furthermore, no significant differences were found for any of the secondary outcome measures between the groups (Table 2).

**Table 2:** Mean difference (95 % CI) between the OsteoACTIVE group and the control group at 6 months and 1-year follow-up

	6 months	1 year	*p*-value
*Quadriceps strength*			
Right peak torque 60° (Nm)	−2.9 (−14.9, 9.0)	6.9 (−5.5, 19.4)	0.692
Left peak torque 60° (Nm)	0.7 (−11.2, 12.6)	6.2 (−6.0, 18.5)	0.792
Right total work 180° (J)	93.4 (−74.9, 261.7)	110.9 (−63.7, 285.8)	0.431
Left total work 180° (J)	43.1 (−125.5, 211.9)	77.8 (−96.1, 251.8)	0.789
*BMD g/cm* ^*2*^			
Lumbar spine (L1-L4)	−.02 (−.07, .02)	−.00 (−.05, .04)	0.818
Hip total	.01 (−.02, .06)	.02 (−.02, .06)	0.615
Femoral neck	.01 (−.02, .05)	.02 (−.01, .06)	0.428
Femoral trochanter	.00 (−.03, .04)	.01 (−.03, .05)	0.954
*Physical capacity*			
FSST (sec)	.4 (−1.8, 2.6)	−.4 (−2.7, 1.8)	0.767
6MWT (m)	41.5 (−1.6, 84.7)	11.3 (−33.1, 84.7)	0.273
Borg’s scale	0.3 (−0.7, 1.4)	0.8 (−0.3, 1.9)	0.280
PASE (0–315)	1.5 (−26.3, 29.4)	24.2 (−4.8, 53.4)	0.328
*SF-36*			
Physical functioning	−2.7 (−10.2, 4.8)	−3.2 (−11.1, 4.6)	0.130
Role limitations-physical	−1.2 (−13.6, 11.1)	3.5 (−9.3, 16.5)	0.109
Bodily pain	−1.6 (−13.2, 9.9)	−3.1 (−15.2, 8.9)	0.545
General health perceptions	−5.2 (−15.1, 4.5)	−1.6 (−11.9, 8.6)	0.708
Vitality	1.0 (−9.2, 11.4)	−.3 (−11.1, 10.4)	0.995
Social functioning	−3.9 (−13.9, 6.1)	5.4 (−4.9, 15.9)	0.404
Role limitations-emotional	−4.5 (−14.3, 5.3)	2.1 (−8.0, 12.4)	0.523
Mental health	.1 (−7.9, 8.2)	2.6 (−5.8, 11.2)	0.937

Values are given as estimated mean group difference (95 % CI)

Linear mixed model (variance component model) with time and time*group as fixed effects, and time as random effect intercept and slope

*CI* confidence interval, *Nm* Newton meter, *J* Joule, *BMD* bone mineral density, *FSST* four square step test, *6MWT* six-minute walk test, *PASE* physical activity scale for the elderly

In general, the estimated means for the OsteoACTIVE group improved or were stable over the follow-up period, except for the decreased quadriceps strength at total work in the right limb and BMD at the lumbar spine and at the femoral trochanter (Table 3). For the control group, the estimated means mainly decreased over the follow-up period, except for some of the variables for quadriceps strength (Table 3).

**Table 3:** Descriptive statistics of the outcome measures at baseline, 6 months and 1-year follow-up for the OsteoACTIVE group and the control group

	OsteoACTIVE group						Control group					
	*N*	*Baseline*	*N*	*6 months*	*N*	*1 years*	*N*	*Baseline*	*N*	*6 months*	*N*	*1 year*
*Quadriceps strength*												
Right peak torque 60° (Nm)	42	95.1 ± 29.1	35	98.1 ± 25.6	31	104.4 ± 22.7	38	95.7 ± 24.7	33	101.0 ± 21.6	32	97.4 ± 24.0
Left peak torque 60° (Nm)	42	87.9 ± 24.6	34	93.3 ± 23.8	31	99.7 ± 25.9	38	88.6 ± 24.5	33	92.6 ± 24.0	32	93.7 ± 25.8
Right total work 180° (J)	42	1181.4 ± 374.4	35	1325.5 ± 368.5	31	1323.6 ± 339.5	38	1182.6 ± 337.3	33	1232.2 ± 318.3	32	1212.7 ± 363.2
Left total work 180° (J)	42	1091.7 ± 344.6	34	1204.7 ± 369.7	31	1236.5 ± 341.7	38	1081.1 ± 341.7	33	1161.5 ± 347.0	32	1164.4 ± 361.1
*BMD g/cm* ^*2*^												
Lumbar spine (L_1_-L_4_)	42	0.951 ± 0.139	35	0.943 ± 0.114	31	0.950 ± 0.110	38	0.955 ± 0.088	33	0.968 ± 0.084	33	0.955 ± 0.095
Hip total	42	0.807 ± 0.079	35	0.822 ± 0.081	31	0.821 ± 0.088	38	0.793 ± 0.099	34	0.806 ± 0.102	33	0.799 ± 0.109
Femoral neck	42	0.781 ± 0.077	35	0.795 ± 0.085	31	0.793 ± 0.081	38	0.767 ± 0.078	34	0.777 ± 0.083	33	0.769 ± 0.094
Femoral trochanter	42	0.644 ± 0.071	35	0.664 ± 0.077	31	0.654 ± 0.085	38	0.641 ± 0.099	34	0.658 ± 0.091	33	0.643 ± 0.103
*Physical capacity*												
FSST (sec)	42	10.9 ± 8.9	34	9.0 ± 3.7	31	7.9 ± 2.5	38	9.9 ± 2.6	34	8.6 ± 1.9	33	8.3 ± 2.5
6 MWT (m)	42	592.8 ± 82.8	34	608.5 ± 94.5	31	596.1 ± 87.3	38	598.2 ± 101.1	34	567.0 ± 83.8	33	584.8 ± 90.5
Borg's scale	42	10.4 ± 2.3	34	10.7 ± 2.7	31	10.8 ± 2.3	38	9.8 ± 2.2	34	10.4 ± 1.9	33	9.9 ± 2.4
PASE (0–315)	42	103.1 ± 56.7	35	123.8 ± 59.4	31	129.1 ± 60.6	38	114.6 ± 58.7	35	122.3 ± 65.0	33	104.8 ± 53.7
*SF-36*												
Physical functioning	42	82.3 ± 16.7	35	84.1 ± 18.7	31	84.5 ± 14.1	38	90.0 ± 11.7	35	86.8 ± 20.0	33	87.7 ± 12.0
Role limitations-physical	42	71.4 ± 31.3	35	81.6 ± 26.8	31	83.8 ± 21.5	38	85.5 ± 21.9	35	82.8 ± 26.4	33	80.3 ± 26.1
Bodily pain	42	70.0 ± 23.1	35	76.0 ± 25.0	31	74.9 ± 22.5	38	77.4 ± 22.3	35	77.6 ± 26.1	33	78.0 ± 27.7
General health perceptions	42	72.0 ± 18.2	35	70.4 ± 22.7	31	72.7 ± 20.8	38	73.9 ± 21.3	35	75.7 ± 21.0	33	74.3 ± 21.2
Vitality	42	56.8 ± 21.1	35	61.2 ± 21.5	31	62.7 ± 18.8	38	57.5 ± 22.7	35	60.1 ± 22.8	33	63.0 ± 23.5
Social functioning	42	82.1 ± 21.0	35	83.2 ± 24.4	31	90.3 ± 19.6	38	87.5 ± 19.9	35	87.1 ± 22.3	33	84.8 ± 22.9
Role limitations-emotional	42	84.7 ± 22.9	35	87.6 ± 22.8	31	90.3 ± 19.6	38	89.9 ± 19.0	35	92.1 ± 14.9	33	88.1 ± 23.6
Mental health	42	77.6 ± 14.9	35	78.7 ± 16.5	31	81.7 ± 13.3	38	76.9 ± 19.5	35	78.5 ± 18.4	33	79.0 ± 19.4

Values are mean ± SD

*CI* confidence interval, *Nm* Newton meter, *J* Joule, *BMD* bone mineral density; *FSST* four square step test, *6MWT* six-minute walk test, *PASE* physical activity scale for the elderly

## Discussion

No significant differences between the OsteoACTIVE group and the control group were found for quadriceps strength, BMD, dynamic balance, walking capacity, physical activity level and QoL over the 1-year follow-up.

Our active rehabilitation programme, OsteoACTIVE, followed the recommended treatment guidelines for postmenopausal women with osteoporosis [42]. The guidelines consisted of weight-bearing activities to improve muscle strength, maintain or increase BMD and balance, prevent falls and fractures, reduce pain and focus on healthy lifestyle, and reduce risk factors for falls. The adherence to the active rehabilitation programme was 87 % where 72 % of the participants fulfilled the a priori goal of 80 % adherence. Our result for adherence is in line with other comparable studies with intervention duration of 6 months [9]. According to Howe et al. [9], adherence is one of the main factors that influence the effectiveness of exercise interventions.

In our study, no significant differences in quadriceps strength between the OsteoACTIVE group and the control group were detected over the 1-year follow-up. However, the OsteoACTIVE group maintained increased quadriceps strength at peak torque 60°/s with 9.8 % (right limb) and 13 % (left limb) at the 1-year follow-up. Also, the control group maintained quadriceps strength, but somewhat less (1.8 % in the right limb and 5.8 % in the left limb). These improvements in quadriceps strength are in line with those reported in the systematic review by deKam et al. [11], who found 3–28 % improvements in quadriceps strength in elderly persons with low BMD who followed an exercise programme with a duration of 12–30 weeks. The explanations for maintained quadriceps strength could be neural adaption and learning effect [43]. Furthermore, it appears that our 6-month active rehabilitation programme had a positive benefit on quadriceps strength without any formal exercise intervention at the 1-year follow-up. Moreover, our intervention seemed to act as a motivation for being more physically active, in light of the increased quadriceps strength that was maintained at the 1-year follow-up. The OsteoINFO with its component of *General exercise guidelines* may have influenced the control group to be more physically active. Despite no significant difference in physical activity level measured with PASE between the groups, the OsteoACTIVE group had an activity level in favour of the control group that was 24.2 points higher at the 1-year follow-up. Our results are supported by RCT conducted by Pereira et al. [44] where they found higher levels of physical activity in the intervention group than the control group 10 years after cessation of the intervention. Despite no significant differences in BMD between our groups, BMD in the spine, total hip, femoral neck and femoral trochanter tended to be stable at the 1-year follow-up for the OsteoACTIVE group (from 1.5 to 1.7 %). This is in accordance with the meta-analysis by Howe et al. [9], who found a positive change of 1 %, but no statistical significant improvements. Previous studies have shown that to gain BMD in postmenopausal women, a period of at least 12–18 months of weight training is recommended [42, 45, 46]. Moreover, we found no clinically significant changes for BMD in the spine or in the hip. Both groups included bisphosphonate users (*n* = 9 in the OsteoACTIVE group, *n* = 14 in the control group), which may have masked the effect of the exercise.

In our study, no statistically significant differences in dynamic balance were found between the groups. An RCT in postmenopausal women with low BMD, conducted by Giaonudis et al. [47], found significant differences between the groups in dynamic balance using the FSST. One explanation of the significant effect on FSST in their study could be the duration of their intervention, which was 18 months, whereas we only studied a 6-month programme. Another explanation could be that our participants were below the cut-off score for multiple fallers, indicating that the FSST was not sensitive to detecting changes over time. Nevertheless, it is unknown whether the FSST is sensitive to change over time [29, 48]. Participants in our study did not differ in walking capacity assessed by the six-minute walk test. This could be due to a ceiling effect already at baseline compared to healthy elderly [49]. In addition, to obtain a clinically significant change, an improvement of 54 m had to be achieved [31]. Mean scores for all subscales of the SF-36 were higher compared to age-matched population scores at baseline [50], and no improvements were found. A meta-analysis by Li et al. [14], has suggested that SF-36 may not be sensitive to detecting changes resulting from an intervention, and that a disease-specific questionnaire could be more appropriate for individuals with low BMD.

Patient education has been demonstrated to improve medication compliance and persistence across a broad range of conditions and disease severity [51], but it remains uncertain whether or not patient education also improves compliance in exercise interventions for osteoporotic patients.

### Strengths and limitations

To our knowledge, this is the first study evaluating the effect of an exercise programme with the use of weight vests in women with low BMD and a previous fracture. Furthermore, our study is one of few studies that have investigated the long-term effect of an exercise intervention among women with low BMD.

We did not monitor number of falls, which constitutes a limitation in this study. Another limitation is the low adherence to reporting physical activity level in the training diary. We may therefore have lost useful information about physical activity habits outside of the group exercise sessions during the 6-month rehabilitation programme. The results could also be a consequence of participants changing their behaviour when entering the study, i.e. the Hawthorne effect [52]. Since only 25 participants met the a priori goal of 80 % adherence to the active rehabilitation programme, we could not perform a per-protocol analysis because it would be underpowered [41]. Furthemore, per protocol analysis may be prone to bias because the reasons for not following the treatment may be attributed to the treatment [41]. Our participants seemed to have better function and QoL compared to previously reported data for healthy elderly persons. Hence, significant improvement in function and QoL may be limited.

## Conclusion

We found no significant effects of a 6-month active rehabilitation programme including a patient education component (OsteoACTIVE) in postmenopausal women with low BMD and a healed wrist fracture over a 1-year follow-up.

## Abbreviations

BMD: bone mineral density
QoL: quality of life
Nm: Newton metres
J: Joules
ICC: intra-class correlation coefficient
BMI: body mass index
DXA: dual-energy x-ray absorptiometry
FSST: four square step test
PASE: physical activity level for the elderly
SF-36: short form 36
6MWT: six-minute walk test
SD: standard deviation
CI: confidence interval
RCT: randomized, controlled trial
